# Induction of Protective CD4^+^ T Cell-Mediated Immunity by a *Leishmania* Peptide Delivered in Recombinant Influenza Viruses

**DOI:** 10.1371/journal.pone.0033161

**Published:** 2012-03-21

**Authors:** Katherine Kedzierska, Joan M. Curtis, Sophie A. Valkenburg, Lauren A. Hatton, Hiu Kiu, Peter C. Doherty, Lukasz Kedzierski

**Affiliations:** 1 Department of Microbiology and Immunology, The University of Melbourne, Parkville, Victoria, Australia; 2 The Walter + Eliza Hall Institute of Medical Research, Parkville, Victoria, Australia; 3 Department of Medical Biology, The University of Melbourne, Parkville, Victoria, Australia; 4 Department of Immunology, St. Jude Children’s Research Hospital, Memphis, Tennessee, United States of America; Federal University of São Paulo, Brazil

## Abstract

The available evidence suggests that protective immunity to *Leishmania* is achieved by priming the CD4^+^ Th1 response. Therefore, we utilised a reverse genetics strategy to generate influenza A viruses to deliver an immunogenic *Leishmania* peptide. The single, immunodominant *Leishmania*-specific LACK_158–173_ CD4^+^ peptide was engineered into the neuraminidase stalk of H1N1 and H3N2 influenza A viruses. These recombinant viruses were used to vaccinate susceptible BALB/c mice to determine whether the resultant LACK_158–173_-specific CD4^+^ T cell responses protected against live *L. major* infection. We show that vaccination with influenza-LACK_158–173_ triggers LACK_158–173_-specific Th1-biased CD4^+^ T cell responses within an appropriate cytokine milieu (IFN-γ, IL-12), essential for the magnitude and quality of the Th1 response. A single intraperitoneal exposure (non-replicative route of immunisation) to recombinant influenza delivers immunogenic peptides, leading to a marked reduction (2–4 log) in parasite burden, albeit without reduction in lesion size. This correlated with increased numbers of IFN-γ-producing CD4^+^ T cells in vaccinated mice compared to controls. Importantly, the subsequent prime-boost approach with a serologically distinct strain of influenza (H1N1->H3N2) expressing LACK_158–173_ led to a marked reduction in both lesion size and parasite burdens in vaccination trials. This protection correlated with high levels of IFN-γ producing cells in the spleen, which were maintained for 6 weeks post-challenge indicating the longevity of this protective effector response. Thus, these experiments show that *Leishmania*-derived peptides delivered in the context of recombinant influenza viruses are immunogenic *in vivo,* and warrant investigation of similar vaccine strategies to generate parasite-specific immunity.

## Introduction

*Leishmania* protozoan parasites shuttle between the sand fly vector, where they multiply as free promastigotes in the gut lumen, and mammalian hosts where they proliferate as obligatory intracellular amastigotes in mononuclear phagocytes [1]. Leishmaniases constitute a family of conditions, with discrete clinical features ranging from cutaneous lesions to a fatal systemic disease. Prevalent in Africa, Latin America, Asia, the Mediterranean basin and the Middle East, leishmaniasis has even been identified in Australia in kangaroos [2]. One of the great neglected diseases, the estimated disease burden places *Leishmania* second in mortality and fourth in morbidity among the tropical infections [3]. Sharp rises in distribution and prevalence have been related to environmental changes and to the migration of non-immune people to endemic areas [4]. The former, in particular, has the potential to expand the geographic span of the vector, thus increasing *Leishmania* transmission to previously unaffected areas [5].

Current treatment is based on chemotherapy, relying on a handful of drugs with serious limitations such as high cost and toxicity, difficult route of administration and lack of efficacy in some endemic areas [6]. Development of a successful vaccine has been a goal for almost a century. There are many barriers to developing an antileishmanial vaccine, but a major issue has been that the traditional approaches have worked poorly. The first generation, whole-cell killed vaccines have been inadequately defined and variable in potency, leading to inconclusive results in field trials. In general, reproducible evidence of protective efficacy has not emerged from clinical trials of first generation leishmaniasis vaccines. The focus is now on the second generation vaccines including genetically modified parasites and defined subunit vaccines, however to date, their efficacy in the field trials has not been reported. Virally vectored vaccines emerged as novel platforms that might address the deficiencies of traditional delivery systems, particularly where cell mediated responses are needed for protection. Influenza viruses are attractive candidates as vaccine vectors, with the approach being tried so far for HIV [7], tuberculosis [8], malaria [9] and cancer [10]. These results point to the value of recombinant influenza vectors for *Leishmania* vaccination. Influenza viruses can be easily manipulated by a reverse genetics strategy [11], which repositions existing immunogenic peptides [12] or inserts additional epitopes into influenza segments [13], [14] to elicit prominent CD8^+^ T cell responses. “Cold-adapted” influenza has been approved for human use (FluMist) [15], and the capacity to readily manipulate the immunogenic peptide in the context of influenza vector makes it easy to apply the vaccine to a number of antigenic candidates. In the present study, we utilised a model of recombinant influenza expressing a single, *Leishmania*-specific LACK_158–173_ (*Leishmania* homologue of receptors for activated C kinase) CD4^+^ T cell peptide. This sequence has been identified by peptide mapping as the major LACK component presented by the I-A^d^ MHC molecule [16]. LACK also has the advantage of being a conserved antigen expressed not only in the sand fly promastigote stage, but importantly, in disease-causing mammalian amastigotes [17], and has been shown to react with sera from patients with cutaneous and visceral leishmaniasis [18]. Here we show that LACK_158–173_ influenza prime/boost immunisation resulted in considerable protection against *Leishmania* in a stringent mouse model of disease, and was associated with increased IFN-γ production by LACK_158–173_-specific CD4^+^ T cells in vaccinated animals.

## Methods

### Mice, viral immunisations and parasite infections

Ethics Statement: Mice were bred at the Walter and Eliza Hall Institute’s animal facility. Animal experiments followed the NHMRC Code of Practice for the Care and Use of Animals for Scientific Purposes guidelines and have been approved by the Walter and Eliza Hall Institute’s Animal Ethics Committee (AEC Projects 2005.012 and 2008.003).

*L. major* virulent clone V121 was derived from the *L. major* isolate LRC-L137 [19] and maintained at 26°C in M199 medium supplemented with 10% (v/v) heat inactivated FBS (Trace Biosciences, NSW, Australia).

The 8 weeks (wk) old naïve BALB/c mice were vaccinated i.p. with 1.5×10^7^ pfu of recombinant influenza A virus in PBS. Mice primed with the PR8-LACK_ins_ virus received a booster injection at 4 wk following priming. PR8 virus controls or PBS controls were included in each experiment. Mice were challenged by intradermal injection of 10^6^ virulent *L. major* live promastigotes at the base of the tail 2 or 6 wk following priming or boosting. Virulent parasites were obtained from a Balb/c mouse infected lesion, which was placed into biphasic blood agar (NNN) medium. Parasites were then passaged in NNN with M119 overlay and left for 6 days when they reached the stationary phase.

Lesion development was monitored using a semi-quantitative scoring of lesion diameter [20]. The following scoring was employed: 0  =  no lesion or minor injection or healing scar; 1  =  small swelling or lesion approximately 1mm in diameter; 2  =  large swelling or lesion 1–5mm in diameter; 3  =  lesion 5–10mm in diameter; 4  =  lesion greater than 10mm in diameter and/or metastasis. Data are expressed as the arithmetic mean ± standard error of the lesion score for the group of mice.

### Generation and titration of recombinant viruses

Recombinant viruses were produced by an 8-plasmid reverse genetics system [11]. The LACK_158–173_ sequence was introduced into the neuraminidase (NA) segment of the H1N1 A/PR8/34 (PR8) virus at position p 42 using either a replacement (LACK_rep_) or insertion strategy (LACK_ins_), and into the H3N2 A/HKx31 (X31) virus at position p 45 using the insertion strategy. The LACK_158–173_ constructs were amplified by PCR from PR8 or X31 genomic template using primers NA-L PR8insF (TTCTCGCCGTCGCTGGAGCATCCGATCGTGGTGTCCGGCAGCTGGGACCAAAACCATACTGGAATATGCAACCAAAACATC) and NA-L PR8insR (GTCCCAGCTGCCGGACACCACGATCGGATGCTCCAGCGACGGCGAGAAACTTCCAGTTTGAATTGAATGGCTAATCC) for PR8-LACK_ins_; NA-L PR8repF (TTCTCGCCGTCGCTGGAGCATCCGATCGTGGTGTCCGGCAGCTGGGACCAAAACATCATTACCTATAAAAATAGCACC) and NA-L PR8repR (as NA-L PR8insR) for PR8-LACK_rep_; NA-L x31p45F (TTCTCGCCGTCGCTGGAGCATCCGATCGTGGTGTCCGGCAGCTGGGACCCCGCGAGCAACCAAGTAATGCCGTGTGAA) and NA-L x31p45R (GTCCCAGCTGCCGGACACCACGATCGGATGCTCCAGCGACGGCGAGAAGGAGTC GCACTCATATTGCTTAAAATGCAATG) for X31-LACK. The regions flanking the LACK epitope within the NA segment are shown in Table S1.

PCR products were digested with *Bsm*B1 or *BsaI* and ligated into pHW2000 vector. The recombinant viruses containing LACK_158–173_ (PR8-LACK_ins_, PR8-LACK_rep_ and X31-LACK) were rescued after transfection of 8 plasmids encoding influenza segments into 293T and MDCK cells. Viruses were amplified in the allantoic cavity of d10 embryonated hen’s eggs and quantified as plaque-forming units (pfu) on monolayers of MDCK cells. The infectious titres of the viruses were 2.6×10^8^, 4.6×10^8^, 2.2×10^8^, 8.4×10^8^ and 7.74×10^8^ pfu/ml for PR8, PR8-LACK_ins_, PR8-LACK_rep_, X31 and X31-LACK, respectively. We ensured that the viral NA construct expressed LACK_158–173_ insert by firstly sequencing the NA plasmid prior to the reverse genetics rescue of the virus, then by sequencing the amplified virus within the allantoic fluid directly from the egg, prior to infection the mice with engineered virus.

### Tissue sampling and cell preparation

Spleens and draining lymph nodes (dLN) were recovered from mice at day 10 following immunisation and at different time-points after *Leishmania* infection (as indicated in the Results). Inguinal dLN were removed after *Leishmania* infection. Spleens and dLN were passed through 40 µm sieves to generate single cell suspension. Parasite burdens were determined by limited dilution analysis [21].

### Cytokine analyses

Cytokine levels were assessed by capture ELISA and Intracellular Cytokine Staining (ICS) [22]. Briefly, for *in vitro* stimulation splenic and dLN cells were resuspended in C-RPMI medium supplemented with 10% FBS, 2-mercaptoethanol, antibiotics and L-glutamine, plated in 48-well plates (Costar, NY, USA) in the presence of 4×10^6^ irradiated (3000 rad) splenocytes and stimulated for 3 days at 37^O^C with LACK_158–173_ peptide or SLA. For *ex vivo* stimulation with the LACK_158–173_ peptide no additional APCs were used, cells were stimulated for 12 h (8 h in the presence of brefeldin A). For ELISA, culture supernatants were collected following LACK_158–173_ or SLA stimulation. U-bottom 96-well plates (Thermo Labsystems, MA, USA) coated with capture antibodies against IL-4, IL-10, IL-12(p 40/p 70) and IFN-γ (BD Pharmingen, CA, USA) were incubated overnight at 4^O^C with serial dilutions of culture supernatants in duplicates, washed and incubated for 2 h at RT with biotin anti-mouse IL-4, IL-10, IL-12(p 40/p 70) and IFN-γ, followed by incubation with streptavidin-horseradish peroxidase (1∶5000). Plates were developed with ABTS in 0.1 M citric acid pH 4.2. Absorbance values were read at 405 nm. Bio-Plex Pro Mouse Cytokine 23-plex assay was performed according to the manufacturers’ instructions (BioRad Laboratories Inc., CA, USA). For ICS, cultured cells were re-stimulated with 50 ng/ml PMA and 500 ng/ml ionomycin in the presence of 2 µg/ml brefeldin A for 4 h at 37^O^C. Cells were stained for surface markers CD4-PE, CD8-PerCP and CD44-APC or FITC, fixed and permeabilised. Cells were stained for either IFN-γ-FITC or IL-4-APC (BD Pharmingen, CA, USA). Cell fluorescence was measured by flow cytometry using a FACSCalibur flow cytometer and data were analysed using BD CellQuest Pro software. LACK_158–173_ peptide was purchased from Auspep (Australia).

### Statistical analyses

Statistical analyses were performed using unpaired t-test [23], [24] provided within GraphPad Prism 5 software and Compare Groups of Growth Curves software package available on the Bioinformatics Division’s website http://bioinf.wehi.edu.au/.

## Results

### Priming with recombinant influenza viruses skews the response to Th1-type

Recombinant PR8 (H1N1) viruses expressing LACK_158–173_ were used to prime susceptible BALB/c mice for the initial characterisation of LACK-specific Th1 and Th2 CD4^+^ T cell responses in comparison to naïve controls. Intraperitoneal priming of mice with influenza does not lead to a productive viral replication, but allows a one-stop growth cycle with full protein production that results in priming of antigen-specific effector T cells in the spleen and the establishment of long-term T cell memory. Although the viruses are replication-competent, they cannot produce infectious progeny due to the absence of the trypsin-like enzyme, which in the wild-type virus cleaves the viral haemagglutinin (HA) in the respiratory tract [25], [26].

**Figure 1:**
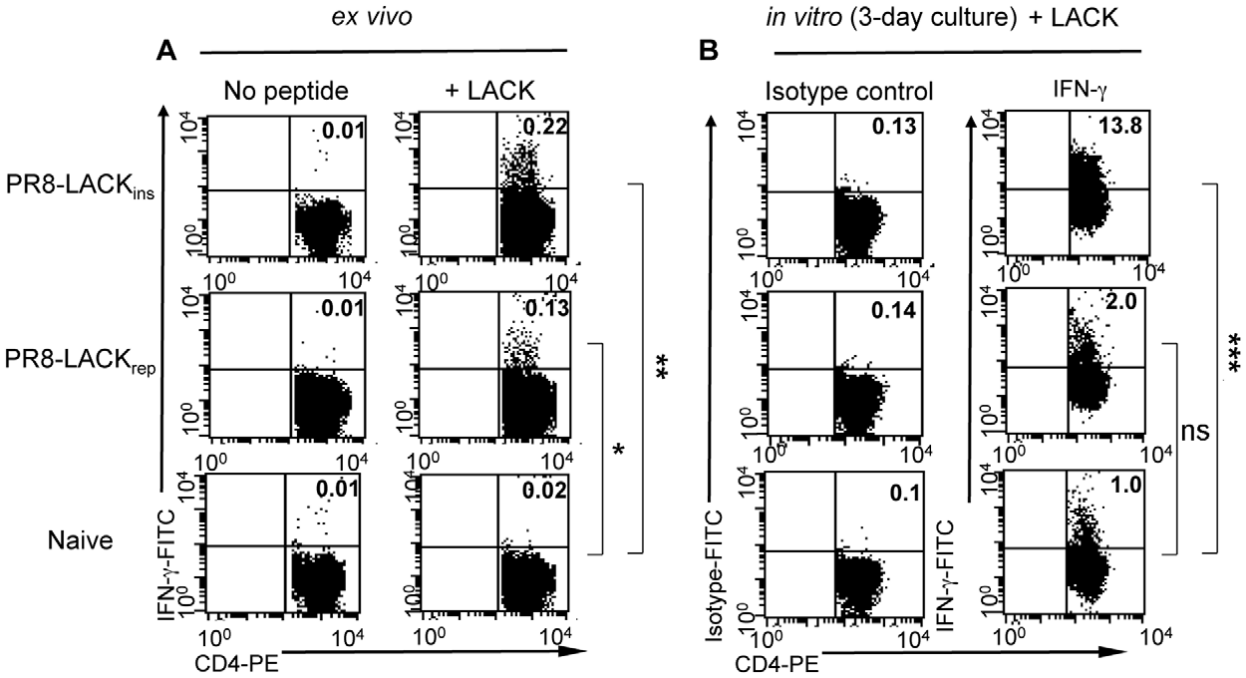
Cytokine production by LACK_158–173_-specific CD4^+^ T cells following influenza-LACK_158–173_ priming. A) *Ex vivo* IFN-γ production by LACK_158–173_-specific CD4^+^ T cells. Cells were obtained from spleens of naïve control mice or PR8-LACK_ins_ and PR8-LACK_rep_ mice on day 10 following priming. Cells were cultured in the absence or presence of LACK_158–173_ peptide for 12 h and assessed for IFN-γ production by ICS. B) *In vitro* IFN-γ production by LACK_158–173_-specific CD4^+^ T cells. Cells were obtained from spleens of naïve control mice or PR8-LACK_ins_ and PR8-LACK_rep_ mice on day 10 following priming. Cells were cultured in the absence or presence of LACK_158–173_ peptide for 72 h followed by re-stimulation with PMA and ionomycin, and assessed for IFN-γ production by ICS. Numbers in the upper right quadrant refer to percentage of CD4^+^ T cells producing IFN-γ (raw data). Cells have been gated on CD4^+^ CD44^+^, for statistical analyses no peptide (panel A) and isotype control (panel B) values were subtracted from the raw values. Representative data from one mouse are shown (n = 3, n refers to number of mice, *p = 0.004, **p = 0.0002, ***p = 0.006).

**Figure 2:**
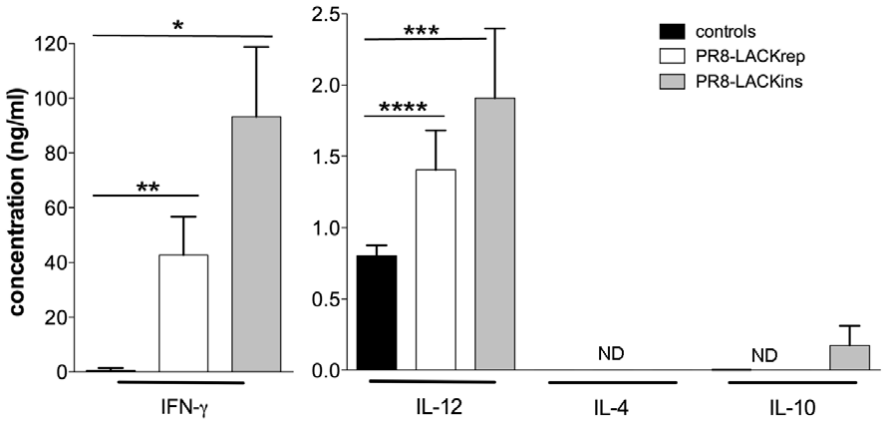
Cytokine profile of LACK_158–175_ specific CD4^+^ T cells. Cells were obtained from spleens of naïve control mice or PR8-LACK_ins_ and PR8-LACK_rep_ mice on day 10 following priming. Cells were cultured in the presence of LACK_158–173_ peptide for 72 h, and culture supernatants were analysed for cytokine production by capture ELISA. Mean, pooled data ± SD are plotted (n = 3, n refers to number of mice) *p = 0.001, **p = 0.003, ***p = 0.009, ****p = 0.01, ND – below detection level.

Two viral constructs expressing the LACK_158–173_ peptide were initially tested for the ability to generate anti-leishmanial responses. Mice were immunised i.p with virus expressing either the LACK peptide sequence inserted within the influenza neuraminidase (NA) gene (LACK_ins_), or with virus in which the LACK sequence replaces a segment of the gene (LACK_rep_) (Table S1). Cells were obtained from spleens at the peak of T cell responses (day 10 (d 10) post-priming) and analysed by a number of assays, including ICS for IFN-γ and IL-4 (Th1 vs Th2), and ELISA to determine the presence of IFN-γ and IL-12 (Th1) versus IL-4 and IL-10 (Th2) in the culture supernatants. Our data show that priming with influenza-LACK_158–173_ elicits LACK-specific CD4^+^ T cells on d 10, suggesting that a single i.p. immunisation can efficiently deliver immunogenic peptides in the context of a recombinant virus. Following 12 h *ex vivo* stimulation with the LACK_158–173_ peptide, a distinct LACK_158–173_^+^CD4^+^ T cell population in the spleens of vaccinated mice produced IFN-γ (Fig. 1A), but no IL-4 (data not shown), indicating that LACK_158–173_ vaccination in the context of influenza induces a Th1-bias. Similar results were obtained after either 24 or 72 h *in vitro* culture with the LACK_158–173_ peptide, when cells were tested for IFN-γ production by ICS assay (Fig. 1B) and by ELISPOT (data not shown). Assessment of multiple cytokine production in the culture supernatant further showed that priming with influenza-LACK_158–173_ induced Th1 responses as the LACK_158–173_ stimulated T cells produced IFN-γ and IL-12, but not IL-4 or IL-10 (very low levels of IL-10 were detected following PR8-LACK_ins_ most likely due to influenza itself, which has been shown to induce IL-10 [27]) (Fig. 2). Interestingly, PR8-LACK_ins_ appeared to be more efficient in the induction of specific CD4^+^ T cells than PR8-LACK_rep_ (Figs. 1, 2), suggesting that the molecular context influences epitope processing and presentation. Accordingly, PR8-LACK_ins_ was used in further experiments. Priming with PR8-LACK_ins_ or PR8-LACK_rep_ did not induce significant IFN-γ production by CD4-negative lymphocytes, which was comparable to the naïve controls (P>0.05) (data not shown). Hence, our results establish that vaccination with LACK_158–173_ peptide in the context of influenza virus delivered by a non-replicative route of immunisation induces antileishmanial LACK_158–173_-specific CD4^+^ T cells polarised towards protective Th1 responses and prompted us to evaluate the protective capacity of these responses.

### Protective efficacy of LACK^+^CD4^+^ T cells in influenza-LACK primed susceptible BALB/c mice

**Figure 3:**
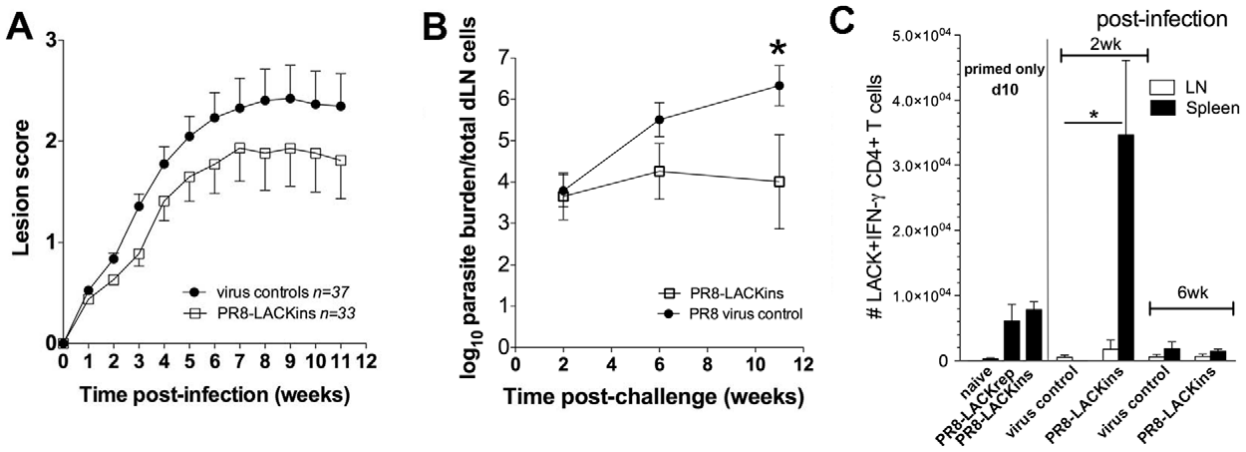
Protective efficacy of influenza-LACK_158–173_ vaccination. A) Lesion scores in mice vaccinated with LACK_ins_ and PR 8 vector controls (pooled data from 2 independent experiments). B) Parasite burdens in mice vaccinated with LACK_ins_ (n = 5), PR 8 vector control (n = 5), *p = 0.02. C) Cytokine production following challenge infection. Spleens and dNL from vaccinated and control mice were assessed by *ex vivo* ICS IFN-γ (*p = 0.006). Mean, pooled data from 2 independent experiments ± SEM are plotted (n = 6 for wk 2, n = 5 for wk 6, n refers to number of mice). Cells have been gated on CD4^+^ CD44^+^.

In order to assess protective efficacy of LACK_158–173_-specific CD4^+^ T cells generated after a single i.p. immunisation with influenza-LACK_158–173_ viruses against *Leishmania* challenge, we performed immunisation trials in Balb/c mice. Animals were primed with recombinant influenza-LACK_158–173_ viruses and were challenged with 10^6^ *L. major* promastigotes at the base of the tail. To monitor the progress of infection, spleens and draining (d)LN were harvested and assessed for parasite burden and cytokine production, and lesion development was monitored weekly. LACK_158–173_-primed susceptible BALB/c mice were partially protected against *Leishmania* infection and showed reduced lesion scores (Fig. 3A) and significantly lower parasite burdens (2–4 log reduction at 11 wk) (Fig. 3B) compared to wild-type (WT) PR 8 virus-primed controls. Even so, while priming led to a substantial decrease in parasite burden, differences in lesion scores did not reach statistical significance. Protection correlated with increased number of IFN-γ producing cells in the spleen of PR8-LACK_ins_ vaccinated mice (and also PR8-LACK_rep_, but to a lesser extent; data not shown) in comparison with the WT PR8 (vector only) primed controls (Fig. 3C). Thus, it seems that LACK_158–173_-specific CD4^+^ T cells generated following immunisation do confer some protection in mice primed with a single dose of replicative influenza-LACK virus delivered bv intraperitoneal route.

### Prime-boost strategy enhances protection and persistence of CD4^+^ T cell responses

**Figure 4:**
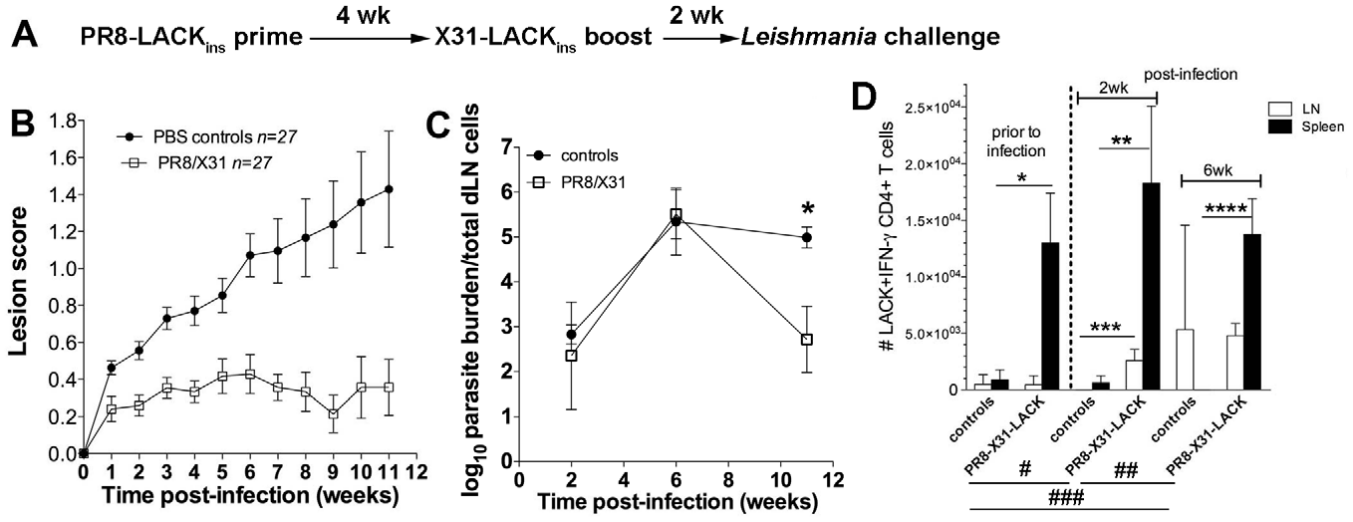
Boosting with serologically distinct recombinant virus increases protective potency of the effector response. A) Prime-boost and challenge strategy. B) Lesion scores in mice primed with PR8-LACK_ins_ and boosted with X31-LACK_ins_ (PR8/X31); and PBS controls. C) Parasite burdens in PR8/X31 vaccinated mice and PBS controls (n = 3 for wk 2 and 6, n = 5 for wk 11), *p = 0.009. D) Cytokine production following challenge. Spleens and dNL from vaccinated and control mice were assessed by *ex vivo* ICS for IFN-γ production. Mean, pooled data ± SEM are plotted (n = 3 for non-challenged mice, n = 5 for wk 2 and 6); *p = 0.02, **p = 0.03, ***p = 0.005, ****p = 0.001, #*p = *0.02, ##*p* = 0.002, ###*p* = 0.03. Cells have been gated on CD4^+^ CD44^+^.

LACK_158–173_-specific CD4^+^ T cells primed by viruses delivered by a non-replicative route of immunisation can provide partial protection against subsequent live parasitic challenge. However, natural infection provides a low level of sustained antigenic stimulation due to parasite persistence, which puts a considerable constraint on vaccine efficacy. Therefore, boosting might be necessary to ensure prolonged protection requiring Th1 responses and induction of high frequencies of specific CD4^+^ T cells. The influenza vector can be easily manipulated by reverse genetics to avoid any pre-existing humoral immunity and allow periodical boosting, an advantage over other virus-based vaccines (eg. vaccinia). To test the suitability of our system for the prime-boost regimen, mice were primed with PR8-LACK_ins_ virus and boosted 4 wk later with X31-LACK_ins_ recombinant influenza. They were then challenged 2 wk following boosting with the parasites and infection was followed for 11 wk. The results showed that immunised mice had significantly (*p*<0.0001) reduced lesion size (Fig. 4B) as well as reduced parasite burdens (*p = *0.009) at the end of the trial compared to PBS controls (Fig. 4C). Protection correlated with a significantly increased number of IFN-γ producing cells in the spleen of vaccinated mice compared to controls, but unlike the situation following priming only, this elevated effector response was still maintained at 6 wk post-challenge indicating that the duration of the protective response has been significantly extended following boosting (Fig. 4D). Moreover, there was a significant expansion of peptide specific CD4^+^ IFN-γ producing cells at the site of infection (dLN). Significantly higher cell numbers were detected in immunised and challenged mice at wk 2 (*p* = 0.02) and wk 6 (*p* = 0.002) compared to pre-challenge levels in unimmunised mice. The expansion of LACK-specific CD4^+^ T cells increased from wk 2 to wk 6 (*p* = 0.03) and was also significantly higher in immunised mice at wk 2 (*p* = 0.005) when compared to non-immunised controls. Although at wk 6 the difference in CD4^+^ IFN-γ producing cells between control and immunised animals was not significantly different, it is worth pointing out that there was only one (out of 3) control mouse that showed specific responses, whereas relatively high numbers of CD4^+^ IFN-γ producing T cells were detected in all immunised mice tested in the assay. No IL-4 production was detected in either the spleen or dLN (data not shown).

### Prime-boost regimen increases longevity of the LACK_158–173_-specific CD4^+^ T cell response

**Figure 5:**
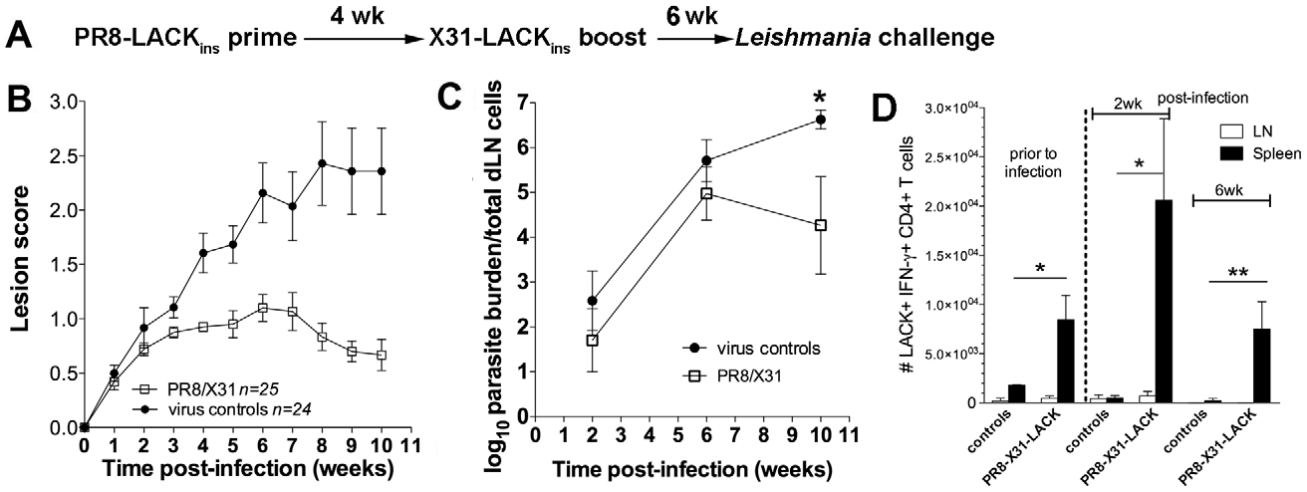
Boosting with serologically distinct recombinant virus increases protective potency and longevity of IFN-γ response. A) Prime-boost with delayed challenge strategy. B) Lesion scores in mice primed with PR8-LACK_ins_ and boosted with X31-LACK_ins_ (PR8/X31); and wild type PR8/X31 virus controls. C) Parasite burdens in PR8/X31 vaccinated mice and virus controls (n = 5 each time point, except n = 4 for wk 10 controls), *p = 0.049. D) Cytokine production following challenge. Spleens and dNL from vaccinated and control mice were assessed by *ex vivo* ICS for IFN-γ production. Mean, pooled data ± SEM are plotted (n = 3 for non-challenged mice, n = 5 for wk 2 and 6; *p = 0.02, **p = 0.01). Cells have been gated on CD4^+^ CD44^+^.

**Figure 6:**
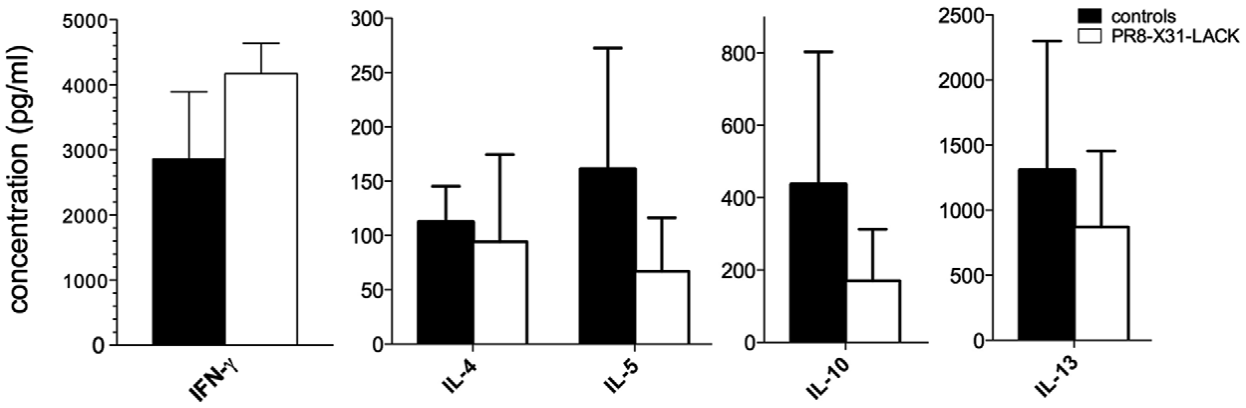
Cytokine production profile of *Leishmania* specific T cells following long-term prime-boost immunisation. Cells were obtained from spleens and dLN of immunised and control mice on wk 2 following challenge. Cells were cultured *in vitro* in the presence of SLA for 72 h, and culture supernatants were analysed for cytokine production. IFN-γ (Th1) levels in splenocytes culture supernatants, and Th2-type cytokine production in dLN on wk 2 post-infection measured by Bio-Plex Pro Mouse Cytokine assay. Mean, pooled data ± SD are plotted (n = 5).

Subsequently, we tested whether our system could be used to trigger long-term protection. Mice were primed with PR8-LACK_ins_ virus (or PR 8 vector alone) and boosted 4 wk later with X31-LACK_ins_ recombinant influenza (or X31 vector alone). Animals were then challenged 6 wk following boosting and infection was followed for 10 wk. Data showed that immunised mice had significantly (*p* = 0.0001) reduced lesion size (Fig. 5B) as well as reduced parasite burdens (*p* = 0.049) at the end of the trial (Fig. 5C). Similarly to the short-term prime-boost experiment, protection correlated with an increased number of LACK_158–173_-specific CD4^+^ IFN-γ producing cells in the spleen of vaccinated mice, with the elevated effector response being maintained at 6 wk post-challenge (Fig. 5D). These levels were significantly higher pre-challenge, at wk 2 and 6 (*p = *0.02, *p* = 0.02 and *p* = 0.01, respectively) in immunised mice compared to controls. There was also a marked increase in numbers of CD4^+^ IFN-γ producing cells in the spleen of vaccinated mice at wk 2 compared to the pre-challenge levels, but this did not reach a level of statistical significance. The CD4^+^ IFN-γ producing profiles in the spleen were similar to those observed previously, but unlike in the previous experiment (Fig. 4D), we did not see an expansion of peptide specific CD4^+^ IFN-γ producing T cells in dLN of vaccinated animals. In addition, we analysed the cytokine profile of the long-term prime-boost and control mice (multiplex analysis of 23 different cytokines and chemokines) in the spleen and dLN at wk 2 and 6 post-challenge, following 3 day *in vitro* stimulation with SLA. Although, the differences did not reach the level of statistical significance, our data show that at wk 2 in the spleen of immunised mice there was a higher level of IFN-γ (Fig. 6). Conversely, the control mice primed with PR 8 and boosted with X31 vectors, displayed higher levels of Th2-type cytokines (IL-4, IL-5, IL-10 and IL-13) in the dLN at wk 2 post-challenge (Fig. 6). At wk 6 post-challenge there was no difference in cytokine production between control and immunised mice (data not shown), indicating that the cytokines may play a crucial role early during the infection. No difference was also observed in the spleens at different time points or in the Th1 (IFN-γ, Il-2, TNF-α and IL-3) cytokine profile (data not shown).

Although we found only trends towards increased Th1 IFN-γ production and trends towards decreased Th2 IL-4, IL-5, IL-10 and IL-13 cytokine production (Fig. 6) in immunised versus control groups when we measured total *L. major*-specific responses, these data further support our argument of altered cytokine milieu following immunisation with a single peptide in the context of an influenza viral vector. As the total antileishmanial responses were assessed by exposure to SLA in a 3-day *in vitro* culture, which allows the amplification of total *L. major* specific responses as well as any LACK-specific responses at equal rates, the expansion of *L. major*-specific responses directed at multiple peptides derived from multiple *L. major* proteins could mask the expansion of single LACK_158–173_ T cell specificity directed at a single epitope. Such equal expansion rates of LACK-specific and *Leishmania-*specific T cells may have not occurred in vivo in immunised versus control mice. 

## Discussion

The development of a safe, effective and affordable antileishmanial vaccine is a critical global public-health priority. No vaccine is currently available and those tested so far have been disappointing in field studies. Thus, alternative priming strategies employing new vectors, delivery systems and adjuvants are urgently needed. Early experimental studies established a dichotomy between Th1-mediated protection and Th2-mediated disease severity. Failure to mount an efficient anti-*Leishmania* Th1 response was shown to cause progressive disease and absence of lesion resolution [28]. However, the Th1/Th2 paradigm has been questioned in recent times since there is accumulating evidence that an early IL-4 response might not be required to promote susceptibility and there are more complexities in the mechanisms responsible for acquired *Leishmania* immunity [29]. Nevertheless, it is generally accepted that an optimal antileishmanial vaccine should, and would trigger a Th1-type response.

Immunisation with LACK has emerged as controversial issue. LACK appears to promote the expansion of IL-4 secreting T cells thus skewing the response towards deleterious Th2 responses [30], [31]. Susceptible BALB/c mice that were made tolerant to LACK had diminished early Th2 responses and were able to develop protective Th1 responses leading to the control of *L. major* infection [32]. Despite its propensity to induce Th2 type immune responses, immunisation with recombinant LACK and IL-12 triggered short term protective responses, but failed to elicit long-term immunity [33]. The prime/boost immunisation with vaccinia virus expressing LACK led to protective immune responses and partial protection in homologous [34] and heterologous [35] challenge systems. So far, the protective efficacy of LACK has been mainly demonstrated in the *L. major* model. Surprisingly, despite its conservation amongst the *Leishmania* species, LACK failed to protect against visceral leishmaniasis, although immunisation induced strong Th1 responses [36]. More recently, a fusion of LACK with HIV-1 TAT transduction domain delivered in dendritic cells has been shown to be superior to immunisation with LACK alone and improved disease outcome.[37] It is unclear at present what would be a good/protective antigen for use in a human vaccine. The LACK antigen was used in our study as a proof of principle to investigate a novel approach to vaccine development.

It is clear that the influenza recombinant virus used in our study triggers protective antileishmanial immunity in the most stringent model of disease, the susceptible BALB/c mouse. In this model, mice do not normally heal, which does not entirely reflect *L. major* infection in humans that usually self-resolve, but due to this reason any protective effect observed in these mice is highly relevant. In addition, not all CL patients self-heal easily, as there is a significant fraction that develops severe disease [38], [39]. The influenza vector provides an ideal delivery for a *Leishmania* vaccine capable of stimulating highly desirable Th1-type T cell responses, and is also an excellent tool for T cell analysis at a high level of resolution. We have demonstrated that a single-dose immunisation with a single, immunodominant epitope induces strong Th1 responses in the context of recombinant influenza vector. These responses provide partial protection in vaccinated animals that is linked to IFN-γ production by epitope specific CD4^+^ T cells. It is interesting to note that following priming only, we observed a discrepancy between the disease manifestation at the site of infection (lesion scores) and the site of parasite clearance (parasite burdens). This lack of correlation might be partially attributed to a greater frequency of regulatory T cells that are generated by infection and accumulate in the dermis where they suppress the ability of the effector T cells to eliminate parasites. This process has been linked to the production of IL-10 [40], a cytokine that is also implicated in the maintenance of parasite persistence [41]. This discrepancy was not observed in our subsequent experiments, where our boosting strategy with serologically distinct recombinant virus, led to a further improvement in the protection against leishmaniasis and increased longevity of IFN-γ responses. Interestingly, the number of LACK_158–173_-specific IFN-γ producing T cells detected after *Leishmania* challenge in the spleen is not increased greatly over pre-challenge levels established after prime-boost with LACK_158–173_ delivered in the context of influenza vectors. These observations are consistent with our previous findings of the staggering stability of secondary influenza-specific memory T cells established after prime-boost scenario [42]. As shown in our previous publication, secondary influenza-specific T cells are extremely stable in the spleen and their numbers do not subside at all until at least d 274 after the secondary boost, for at least some epitopes. In addition, approximately 50% of those T cells on d 274 after secondary boost retain their CD62L^low^ effector phenotype, thus are strategically positioned and ready to fight the recurrent infection. Such great stability of memory T cells established after influenza priming and boosting further confirms the beneficial use of the influenza vector for eliciting long-term antileishmanial T cell responses. Furthermore, as previously observed for the influenza system [43] although T cells can expand following a second viral exposure, interestingly, tertiary exposure to the antigen appears to have a minimal, further effect. Thus, it is not surprising that LACK_158–173_-specific CD4^+^ T cell numbers are not increased greatly after the challenge (tertiary antigen exposure). Part of the reason for this may be that *Leishmania* challenge of H1N1-LACK_158–173_→H3N2-LACK_158–173_ primed-boosted mice is substantially controlled early after infection, though it is also possible that there may be inherent physiological maxima that ultimately limit the size of these responses in lymphoid tissue.

Protection observed in our system is linked to the high numbers of LACK_158–173_-specific IFN-γ producing CD4^+^ T cells induced in the spleens. In all experiments, these levels are significantly higher than those observed in control, non-immunised animals. Moreover, the levels of LACK_158–173_-specific IFN-γ producing CD4^+^ T cells are markedly although not significantly higher compared to the pre-challenged levels, which is consistent in all our experiments (cell percentages are shown in Fig. S3). As discussed above these cells are either stable and do not subside or the expansion of this pool of cells was more pronounced at an earlier time point (eg. wk 1), hence at wk 2 their post-challenge levels are not significantly greater than pre-challenge levels. At the same time, our prime-boost approach (Fig. 4) shows expansion of LACK_158–173_-specific IFN-γ producing CD4^+^ T cells in dLN of immunised mice, which at wk 6 are still expanding in dLN, although this effect is not observed in our delayed challenge experiment (Fig. 5). Interestingly, the levels of LACK-specific CD4^+^ T cells have not expanded dramatically following boosting compared to priming only, however, the protective efficacy of prime-boost approach was significantly better. It remains unclear why the levels of IFN-γ producing LACK-specific cells have not increased, but it is well established that CD4^+^ memory T cells, proliferating in response to restimulation, divide for a shorter period of time and as a consequence do not expand significantly following recall response [44]. Importantly, there is generally enhanced avidity (i.e. quality) of boosted T cell responses, thus the prime-boost vaccination could have generated a higher ratio of multifunctional Th1 CD4^+^ T cells, which have been shown to be more efficient effector cells [45]. In this case, a more efficacious immune response could be achieved without further expansion of cell numbers. Additionally, the low-avidity CD4^+^ T cell responders have been shown to have diminished survival capacity and are rapidly eliminated [46]. Therefore, the pool of primed CD4-specific T cells could have been initially reduced and then expanded following boosting to seemingly the same magnitude, but with much better quality of response.

In addition, the magnitude of response is controlled by the duration of secondary stimulus [47] and it has also been observed that CD4^+^ memory cells can decline in numbers in mice [48], which in turn can lead to smaller numbers following recall. It has also been reported that the efficacy of T cell responses correlates with the number of memory T cells in peripheral sites rather than in secondary lymphoid organs [49]. It is therefore possible that there were significantly larger numbers of effector memory T cells in the peripheral tissue, which allowed them to respond more efficiently o *Leishmania* challenge. The above scenarios might be responsible for the apparent lack of expansion of LACK-specific CD4^+^ T cells following boosting.

The analysis of cytokine profile following SLA stimulation indicates a clear trend towards elevated levels of detrimental Th2-type cytokines in non-immunised controls, which can be linked to the lack of protection. Our previous work [22] showed that the most striking differences in cytokine production between immunised and control animals occur at wk 1 post-challenge. It is therefore, likely that higher levels of Th2 cytokines at an early time point were decisive in directing the response towards non-protective Th2 response in control animals, but not in the immunised animals which could mount protective Th1 response even in the apparent absence of elevated levels of Th1-type cytokines at the early time point post-challenge.

Although protection has been observed, it needs to be acknowledged that high-dose needle challenge has some drawbacks when it comes to linking the correlates of protective immunity with vaccine efficacy against natural infection. Peters *et* *al.* [50] have demonstrated that sand fly transmission of parasites abrogates vaccine-induced protective immunity. While mice vaccinated with killed parasites were refractory to a needle challenge, they were susceptible to the sand fly inoculum implying that the responses in vaccinated mice required for protection were either not generated or not maintained. On the other hand, mice that healed the primary lesion were protected against sand fly challenge, and the rapidity of the response suggested that the protective response was not derived from the central memory, but rather from an effector pool of T cells that could have been maintained by the persistent parasites. Therefore, the influenza-vectored vaccine would need to be tested in a natural model of infection to assess its full efficacy.

The influenza virus itself skews the response towards highly desirable Th1-type, but importantly, it also engages host pattern recognition receptors, such as Toll receptors [51], stimulating the innate immune system which in turn enhances the adaptive responses triggered by the vaccine. The influenza system also offers the prospect of inducing cytotoxic CD8^+^ T cells, which contribute to the disease outcome. Initially, antileishmanial CD8^+^ T cells were thought to play a role only during re-infection [52], however, they were subsequently shown to be crucial in controlling the primary infection by skewing the responses towards Th1 [53], [54]. Beside cytokine production, CD8^+^ T cells are also thought to participate in controlling the infection through cytotoxic mechanisms, but the exact route of CD8^+^ T cell activation in leishmaniasis is still not known. Since the parasites reside in a parasitophorous vacuole inside the host macrophages it is not entirely clear how these cells present antigen through MHC I [55], [56]. The most likely mechanism is cross-presentation, which has been well documented for macrophages and DCs [57], [58], but to date has not been demonstrated in *Leishmania* infection. We analysed the CD8^+^ contribution in the spleen and dLN at wk 2 and 6 post-challenge, following 3 day *in vitro* stimulation with SLA. Although, the differences did not reach the level of statistical significance (Fig. S1), our data show that at wk 2 in the spleen of immunised mice there were higher numbers of antigen experienced IFN-γ+ CD4^+^ and CD8^+^ T cells compared to controls. In particular, the contribution of CD8^+^ T cells was substantially greater to that of CD4^+^ T cells, which is consistent with their reported role in the rapid resolution of secondary infection in re-challenged and vaccinated mice [59]. However, as the vaccine does not trigger LACK-specific CD8^+^ T cells, those cells would not be elicited after either a prime or boost with influenza-LACK vaccine. Thus, we expect that CD8^+^ T cell responses observed in our experiment are broadly *Leishmania*-specific as they respond to SLA stimulation. Therefore, the delivery of leishmanial epitopes in the context of influenza vector can facilitate the class I presentation in addition to class II presentation of a recombinant peptide. The capacity to readily manipulate the immunogenic peptide in the context of influenza vector makes it easy to adapt the vaccine for a number of candidate antigens. As the vector can be manipulated by reverse genetics to include multiple epitopes, this approach has the capacity to generate broad protective responses with the potential to overcome HLA restriction and provide immunological coverage to the broader host population. Regions of up to 240 aa have been successfully cloned into the NA region and viruses rescued [7], thus providing scope for insertion of polytopic fragments. Alternatively, combinations of recombinant vectors expressing single epitopes could be used to increase the efficacy of the vaccine.

A potential obstacle for a vaccine based on a viral vector is safety. However, there is already a precedent in using live, temperature sensitive influenza virus in a FluMist vaccine approved by the FDA (live-attenuated, intranasal vaccine) [60]. Moreover, the insertion of foreign sequences into the NA segment results in a virus that can express the recombinant peptide as part of the NA protein, but cannot release virus progeny from infected cells [61]. In our study, insertion of the 16 aa LACK_158-173_ peptide into the viral NA led to a decrease in viral replication on d 3 after intranasal inoculation (Fig. S2) and mild symptoms of influenza infection (modest weight loss) when compared to mice infected with WT PR8 virus (50 pfu) which showed severe signs of infection (loss of body weight, ruffled fur, unresponsiveness). Furthermore, a reversion to the replication-competent NA-deficient virus via recombination with a WT virus is highly unlikely.

Vaccination with a virally-vectored vaccine triggers an immune response against the recombinant antigen as well as the vector. Although a homologous boost approach is feasible, it usually leads to diminished responses and affects the immunogenicity of a vaccine as in a case of vaccinia-based vaccines [62]. Due to annual changes (antigenic drift) within the NA and HA segments, antibody protection is specific to a particular strain of influenza. Thus, antibody-based anti-influenza vaccines need to be updated annually. Hence, it is highly unlikely that there will be pre-existing humoral immunity to the particular viral strain used as a vaccine vector. Furthermore, the vector can be manipulated by reverse genetics to avoid any possible neutralising humoral immunity and thus allow periodical boosting. Our data indicate that boosting with serologically different recombinant influenza increased the immunogenicity of the vaccine, resulted in significantly better host protection and increased the longevity of the IFN-γ-driven immune response.

An additional advantage of using influenza-vectored vaccines is that they can be used as effective vaccines against seasonal influenza viruses. Recent study using recombinant influenza expressing West Nile Virus domain III of glycoprotein E [63], demonstrated that in addition to inducing protective levels of antibodies and CD4+ T cells directed against the West Nile Virus, vaccination also induced humoral responses against the vector at protective levels. Therefore, the influenza vector system might be a suitable platform for development of bivalent vaccine against leishmaniasis and influenza.

Vaccination is by far the most cost effective means of control for infectious diseases such as leishmaniasis and the recombinant influenza viruses might provide an attractive alternative vector for its delivery. Although LACK might not be the eventual vaccine molecule, the successful generation of recombinant influenza viruses expressing *Leishmania*-specific epitopes and the demonstration that protection can be induced in the most susceptible animal model of disease, provide proof of concept that an influenza-vectored vaccine is feasible and worthy of future development/investigation.

## Supporting Information

Figure S1 **CD8^+^ T cells contribution to antileishmanial immunity following prime-boost immunization regimen.** Cells were obtained from spleens of immunised and control mice on wk 2 following challenge. Cells were cultured *in vitro* in the presence of SLA for 72 h, and restimulated with PMA and ionomycin in the presence of brefeldin A for 4 h at 37^o^C, followed by *in vitro* ICS for IFN-γ production.
(TIF)

Figure S2 **Viral replication kinetics following intranasal infection with the WT PR8 and recombinant PR8-LACK viruses.** Naïve mice were infected with either WT PR8 or the mutant PR8-LACK_ins_ virus. Lungs were sampled at days 3 and 9 after infection and homogenized for titration in a standard plaque assay. The results are log_10_ pfu per lung. Individual mouse (symbols) and a mean value (line) are shown. *p<0.05.
(TIF)

Figure S3 **Percentages of LACK+ IFN-γ producing CD4^+^ T cells.** A) Primed only mice; B) Short-term primed-boosted mice; C) Long-term primed-boosted mice. Mean, pooled data ± SEM are plotted (n numbers as per legends to Fig. 3, 4 and 5, respectively).
(TIF)

Table S1 Amino acid sequences of the regions flanking LACK_158-173_ within the influenza neuraminidase of the H1N1 A/PR8/34 (PR8) and the H3N2 A/HKx31 (X31) virus (LACK aa sequence underlined).
(DOC)
