# Supplementary figures and images for: Induction of Protective CD4+ T Cell-Mediated Immunity by a Leishmania Peptide Delivered in Recombinant Influenza Viruses
Source: PLoS One. 2012 Mar 21;7(3):e33161. doi: 10.1371/journal.pone.0033161 (PMC3310046; doi:10.1371/journal.pone.0033161)

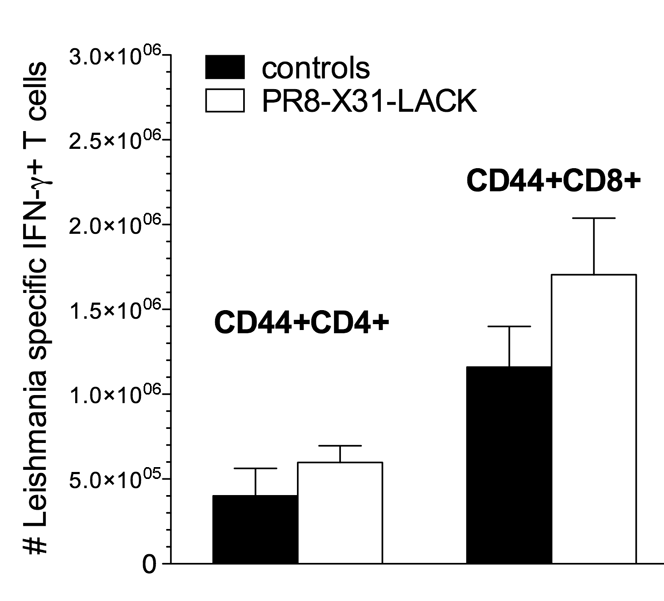

Supplement: Figure S1 — CD8+ T cells contribution to antileishmanial immunity following prime-boost immunization regimen. Cells were obtained from spleens of immunised and control mice on wk 2 following challenge. Cells were cultured in vitro in the presence of SLA for 72 h, and restimulated with PMA and ionomycin in the presence of brefeldin A for 4 h at 37oC, followed by in vitro ICS for IFN-γ production. (TIF) [file pone.0033161.s001.tif]

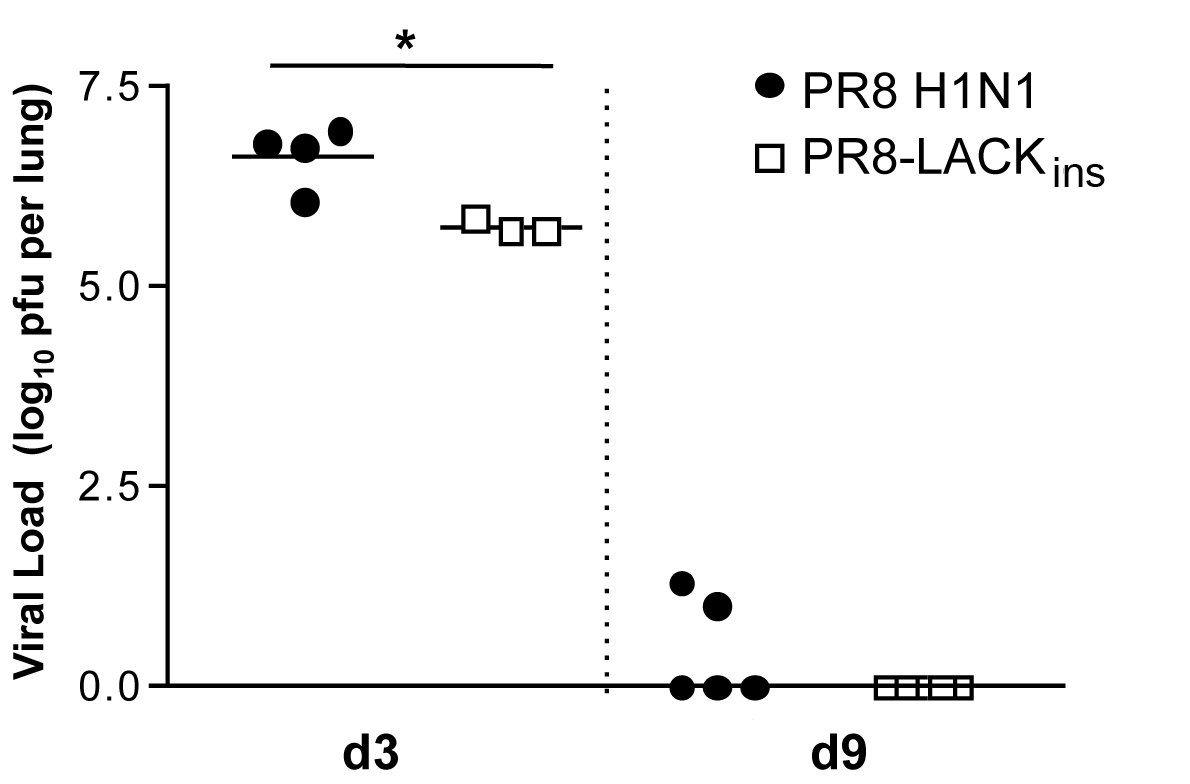

Supplement: Figure S2 — Viral replication kinetics following intranasal infection with the WT PR8 and recombinant PR8-LACK viruses. Naïve mice were infected with either WT PR8 or the mutant PR8-LACKins virus. Lungs were sampled at days 3 and 9 after infection and homogenized for titration in a standard plaque assay. The results are log10 pfu per lung. Individual mouse (symbols) and a mean value (line) are shown. *p<0.05. (TIF) [file pone.0033161.s002.tif]

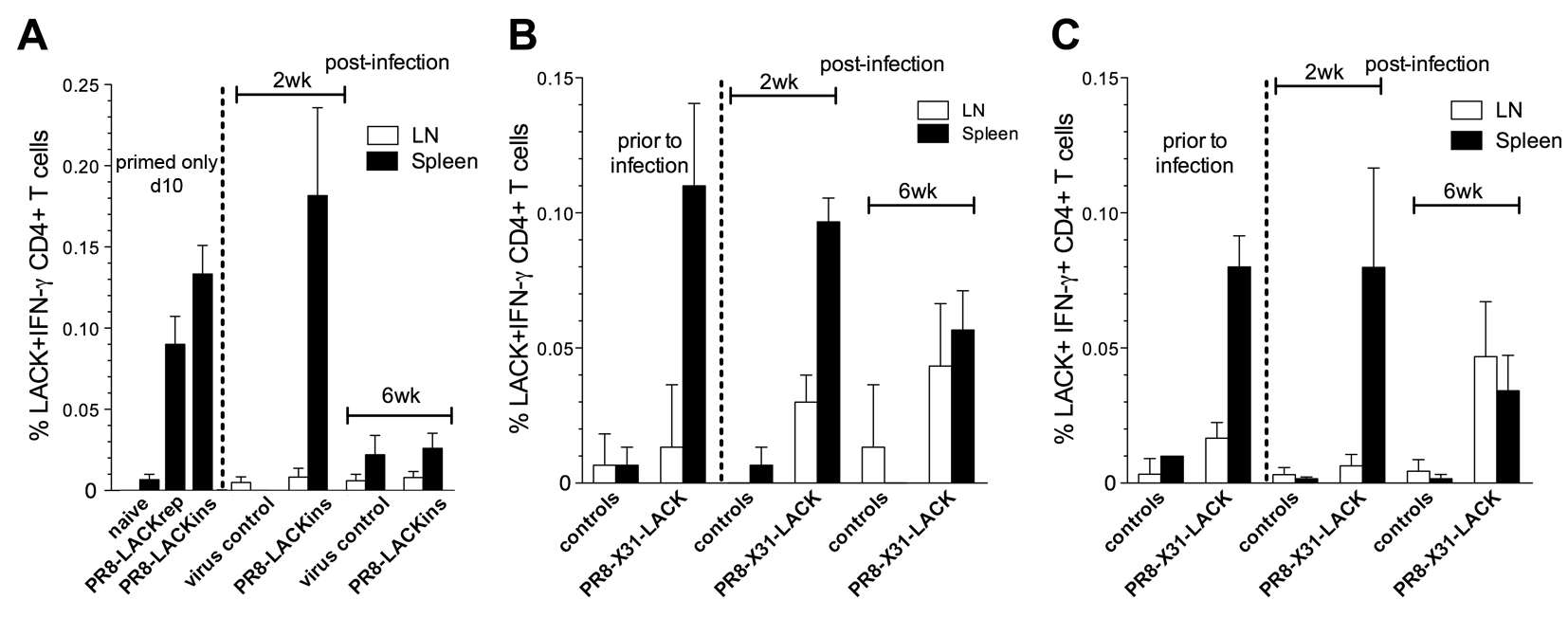

Supplement: Figure S3 — Percentages of LACK+ IFN-γ producing CD4+ T cells. A) Primed only mice; B) Short-term primed-boosted mice; C) Long-term primed-boosted mice. Mean, pooled data ± SEM are plotted (n numbers as per legends to Fig. 3, 4 and 5, respectively). (TIF) [file pone.0033161.s003.tif]
