# Supplementary material for: Induction of Protective CD4+ T Cell-Mediated Immunity by a Leishmania Peptide Delivered in Recombinant Influenza Viruses
Source: PLoS One. 2012 Mar 21;7(3):e33161. doi: 10.1371/journal.pone.0033161 (PMC3310046; doi:10.1371/journal.pone.0033161)
Supplement: Table S1 — Amino acid sequences of the regions flanking LACK158-173 within the influenza neuraminidase of the H1N1 A/PR8/34 (PR8) and the H3N2 A/HKx31 (X31) virus (LACK aa sequence underlined). (DOC) [file pone.0033161.s004.doc]

| **Construct** | **Amino Acid Sequence** | **Position** |
| --- | --- | --- |
| LACK_158-173_ | FSPSLEHPIVVSGSWD |  |
| PR8 NA-LACK_ins_ | MNPNQKITTIGSICLVVGLISLILQIGNIISIWISHSIQTGS**FSPSLEHPIVVSGSWD**QNHTGICNQNIITYKNSTWVKDTTSVITGNSSLCPIRGWAIYSKDNSIRIGSKGDVFVIREPFISCSHLECRTFFLTQGALLNDKHSNGTVKDRSPYRALMSCPVGEAPSPYNSRFESVAWSASACHDGMGWLTIGISGPDNGAVAVLKYNGIITETIKSWRKKILRTQESECACVNGSCFTIMTDGPSDGLASYKIFKIEKGKVTKSIELNAPNSHYEECSCYPDTGKVMCVCRDNWHGSNRPWVSFDQNLDYQIGYICSGVFGDNPRPEDGTGSCGPVYVDGANGVKGSYRYGNGVWIGRTKSHSSRHGFEMIWDPNGWTETDSKFSVRQDVVAMTDWSGYSGSFVQHPELTGLDCMRPCFWVELIRGRPKEKTIWTSASSISFCGVNSDTVDWSWPDGAELPFSIDK | 42 |
| PR8NA-LACK_rep_ | MNPNQKITTIGSICLVVGLISLILQIGNIISIWISHSIQTGS**FSPSLEHPIVVSGSWD**QNIITYKNSTWVKDTTSVILTGNSSLCPIRGWAIYSKDNSIRIGSKGDVFVIREPFISCSHLECRTFFLTQGALLNDKHSNGTVKDRSPYRALMSCPVGEAPSPYNSRFESVAWSASACHDGMGWLTIGISGPDNGAVAVLKYNGIITETIKSWRKKILRTQESECACVNGSCFTIMTDGPSDGLASYKIFKIEKGKVTKSIELNAPNSHYEECSCYPDTGKVMCVCRDNWHGSNRPWVSFDQNLDYQIGYICSGVFGDNPRPEDGTGSCGPVYVDGANGVKGFSYRYGNGVWIGRTKSHSSRHGFEMIWDPNGWTETDSKFSVRQDVVAMTDWSGYSGSFVQHPELTGLDCMRPCFWVELIRGRPKEKTIWTSASSISFCGVNSDTVDWSWPDGAELPFSIDK | 42 |
| X31NA-LACK_ins_ | MNPNQKIITIGSVSLTIATVCFLMQIAILVTTVTLHFKQYECDS**FSPSLEHPIVVSGSWD**PASNQVMPCEPIIIERNITEIVYLNNTTIEKEICPKVVEYRNWSKPQCQITGFAPFSKDNSIRLSAGGDIWVTREPYVSCDHGKCYQFALGQGTTLDNKHSNDTIHDRIPHRTLLMNELGVPFHLGTRQVCIAWSSSSCHDGKAWLHVCITGDDKNATASFIYDGRLVDSIGSWSQNILRTQESECVCINGTCTVVMTDGSASGRADTRILFIEEGKIVHISPLSGSAQHVEECSCYPRYPGVRCICRDNWKGSNRPVVDINMEDYSIDSSYVCSGLVGDTPRNDDRSSNSNCRNPNNERGNQGVKGWAFDNGDDVWMGRTISKDLRSGYETFKVIGGWSTPNSKSQINRQVIVDSDNRSGYSGIFSVEGKSCINRCFYVELIRGRKQETRVWWTSNSIVVFCGTSGTYGTGSWPDGANINFMPI | 45 |

LACK_rep_ – replacement strategy

LACK_ins_ – insertion strategy
